# Restraint Stress-Induced Immunosuppression Is Associated with Concurrent Macrophage Pyroptosis Cell Death in Mice

**DOI:** 10.3390/ijms241612877

**Published:** 2023-08-17

**Authors:** Chi-Cheng Li, Rina Munalisa, Hsuan-Yun Lee, Te-Sheng Lien, Hao Chan, Shih-Che Hung, Der-Shan Sun, Ching-Feng Cheng, Hsin-Hou Chang

**Affiliations:** 1 Department of Hematology and Oncology, Buddhist Tzu Chi General Hospital, Hualien 970, Taiwan; kevinlcc1234@gmail.com; 2 Center of Stem Cell & Precision Medicine, Hualien Tzu Chi Hospital, Hualien 970, Taiwan; 3 Department of Molecular Biology and Human Genetics, Tzu-Chi University, Hualien 970, Taiwan; 108727110@gms.tcu.edu.tw (R.M.); 109727102@gms.tcu.edu.tw (H.-Y.L.); alan211@mail.tcu.edu.tw (T.-S.L.); chanhao1011@gmail.com (H.C.); 102353113@gms.tcu.edu.tw (S.-C.H.); dssun@mail.tcu.edu.tw (D.-S.S.); 4 Department of Pediatrics, Taipei Tzu Chi Hospital, Buddhist Tzu Chi Medical Foundation, Taipei 231, Taiwan; chengcf@mail.tcu.edu.tw; 5 Institute of Biomedical Sciences, Academia Sinica, Taipei 115, Taiwan

**Keywords:** restraint stress, immunosuppression, macrophage, cell death, pyroptosis, psychological stress, ambient cold exposure, intravenous immunoglobulin

## Abstract

Psychological stress is widely acknowledged as a major contributor to immunosuppression, rendering individuals more susceptible to various diseases. The complex interplay between the nervous, endocrine, and immune systems underlies stress-induced immunosuppression. However, the underlying mechanisms of psychological-stress-induced immunosuppression remain unclear. In this study, we utilized a restraint stress mouse model known for its suitability in investigating physiological regulations during psychological stress. Comparing it with cold exposure, we observed markedly elevated levels of stress hormones corticosterone and cortisol in the plasma of mice subjected to restraint stress. Furthermore, restraint-stress-induced immunosuppression differed from the intravenous immunoglobulin-like immunosuppression observed in cold exposure, with restraint stress leading to increased macrophage cell death in the spleen. Suppression of pyroptosis through treatments of inflammasome inhibitors markedly ameliorated restraint-stress-induced spleen infiltration and pyroptosis cell death of macrophages in mice. These findings suggest that the macrophage pyroptosis associated with restraint stress may contribute to its immunosuppressive effects. These insights have implications for the development of treatments targeting stress-induced immunosuppression, emphasizing the need for further investigation into the underlying mechanisms.

## 1. Introduction

The effects of psychological stress on the immune system and its ability to trigger immunosuppression have been extensively examined [1,2,3,4,5]. It is well-established that chronic or prolonged psychological stress can lead to dysregulation of immune function, resulting in increased susceptibility to infections, delayed wound healing, and heightened risk of autoimmune and inflammatory diseases. The intricate interplay between the nervous, endocrine, and immune systems is involved in the mechanisms of psychological-stress-induced immunosuppression. However, the specific details of these mechanisms remain elusive.

The restraint stress model is a well-recognized tool to study psychological-stress-related physiological, behavioral, and biochemical changes in mice [6,7,8,9]. It has been shown that chronic restraint stress can lead to immunosuppression [10,11,12,13]. However, the impact of psychological alterations linked to acute restraint stress on the induction of an immunosuppressive response remains unclear. We have previously used a modified acute restraint stress mouse model by administering the non-absorbable dye Evans blue via gavage, which does not normally appear in the circulation when orally fed [14,15,16,17]. Through the analysis of plasma levels of Evans blue, a substance that leaks from the gastrointestinal (GI) tract, we can assess the extent of stress-induced GI leakage. This measurement has the potential to provide real-time indications of stress levels in living animals. With the assistance of this useful tool, we would like to address acute restraint-stress-associated immunosuppression.

In this study, we aim to compare restraint-stress-induced immunosuppression with two other established models: intravenous immunoglobulin (IVIg) administration and ambient cold exposure [18,19]. IVIg is a mixture of human antibodies and a clinically used drug in immunoglobulin therapy to treat inflammatory and autoimmune diseases, such as immune thrombocytopenia (ITP), Kawasaki disease, systemic lupus erythematosus, dermatomyositis, neurologic disorders, and even COVID-19 infections [20,21,22,23,24,25,26,27,28,29,30,31,32,33]. IVIg-induced immunosuppression refers to the suppression of the immune system following the administration of IVIg, which contains high levels of antibodies; however, its mechanism is still not fully understood [14]. Similarly, ambient cold exposure also leads to immunosuppression with complicated regulation [18]. In our findings, we observed that IVIg and ambient cold exposure induce immunosuppression through a P-selectin-dependent pathway [18,19], while acute restraint stress is not associated with this pathway. In addition, here we found that macrophage cell death is connected to immunosuppression caused by restraint stress, while it is not associated with immunosuppression induced by IVIg or ambient cold exposure.

These findings imply that the inhibition of macrophages and the induction of macrophage cell death might contribute to the immunosuppressive effects of restraint stress. However, the exact mechanism underlying macrophage-suppression-induced immunosuppression remains unclear and is rarely discussed in the context of pathophysiological changes in such stress. Existing evidence indicates that macrophage suppression is associated with several anti-inflammatory and immunosuppressive conditions. For example, studies conducted on diabetic animals revealed that insulin deficiency resulted in immunosuppressive effects, which were evident through the impairment of macrophage phagocytosis and cytokine production. These effects were subsequently restored upon administration of insulin [34,35,36]. In vivo depletion of macrophages using clodronate liposome treatments has been shown to suppress various experimental inflammatory diseases [37,38,39,40]. Despite these observations, the specifics of macrophage suppression and depletion in restraint-stress-induced immunosuppression remain largely unknown.

In our study, we also discovered a connection between pyroptosis, one of the regulated cell death pathways [41,42,43,44,45], and restraint-stress-induced immunosuppression. Pyroptosis is a highly inflammatory type of programmed cell death that occurs in response to infection or cellular damage [44,45,46]. It involves the activation of the inflammasome and its crucial component caspase-1, which initiates pyroptosis [44,45,46]. Certain treatments, such as canonical inflammasome NLRP3 inhibitors OLT1177 and caspase-1 inhibitor Z-WEHD-FMK, have been reported to block the cascade of events leading to pyroptosis and subsequent inflammation [41,42,43,46,47,48]. However, the specific role of macrophage pyroptosis in restraint-stress-induced immunosuppression remains unclear and warrants further investigation.

Taken together, our findings indicate that macrophage pyroptosis cell death is linked to the immunosuppression induced by restraint stress. In the following sections, we will delve into potential regulations that underlie this stress-induced immunosuppressive response.

## 2. Results

### 2.1. Restraint Stress Resulted in the Development of Anxiety-like Behaviors in C57Bl/6J Mice

**Figure 1:**
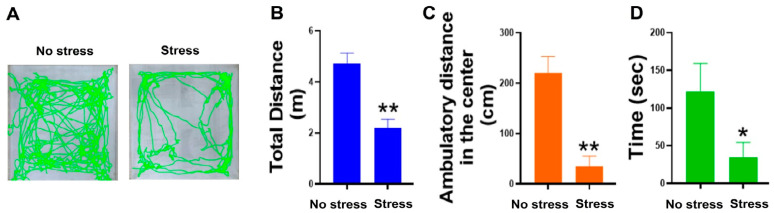
Restraint stress resulted in the development of anxiety-like behaviors in mice. During the 20 h stress procedure, both the no stress and stress groups were deprived of access to food and water. After the termination of restraint stress, both the unstressed and stressed groups of mice were given access to food and water for a period of 2 h to restore their resources. Subsequently, the OFT was conducted. Representative video tracking images captured during a 5-min OFT are presented (**A**). A comparison was made between the control group (no stress) and the restraint stress group (stress) for total distance traveled (**B**), distance traveled in the central zone (**C**), and time spent in the central area (**D**). The number of samples used for analysis was 3 (*n* = 3). * indicates statistical significance at *p* < 0.05, ** indicates statistical significance at *p* < 0.01, when compared to their respective no stress groups.

Restraint stress exposes experimental mice to both physiological and psychological stresses. To investigate the potential involvement of psychological stress in the Evans-blue-fed restraint stress mouse model, we conducted the open field test (OFT) mouse behavior test according to previously reported methods [49,50]. The results revealed that mice subjected to Evans-blue-fed restraint stress treatment exhibited anxiety-related behavior, providing evidence of psychological stress’s contribution to this model (Figure 1). These findings are consistent with numerous previous reports that have identified psychological stress as one of the major factors associated with restraint stress animal models [6,7,8,16,51,52,53,54,55,56,57,58,59,60,61,62]. Additionally, as psychological-stress-induced immunosuppression is rarely discussed, this model is suitable to address this question.

### 2.2. The Mechanism of Immunosuppression Differs between Restraint Stress, Intravenous Immunoglobulin (IVIg), and Ambient Cold Exposure in Mice

**Figure 2:**
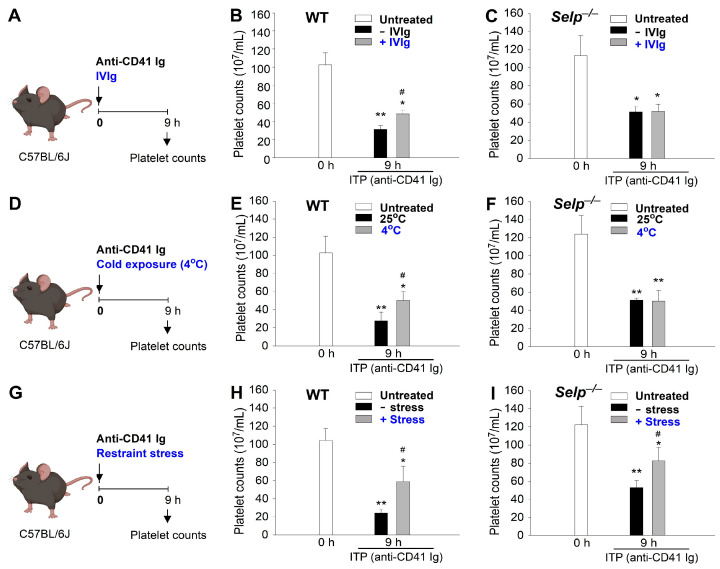
Treatments of IVIg, cold exposure, and restraint-stress-induced immunosuppression as indicated by the amelioration of anti-CD41 Ig (an antiplatelet Ig)-induced immune thrombocytopenia (ITP). (**A**–**C**) IVIg (+IVIg) vs. vehicle (−IVIg) treatments. (**D**–**F**) Cold exposure (4 °C) vs. room temperature (25 °C). (**G**–**I**) Restraint stress (+stress) vs. no stress (−stress). (**A**,**D**,**G**) Experiment outlines. (**B**,**E**,**H**) Experiments employed wild-type (WT) mice. (**C**,**F**,**I**) Experiments employed P-selectin deficient mice (*Selp^−/−^*) mice. * *p* < 0.05, ** *p* < 0.01, vs. respective 0 h groups; # *p* < 0.05, vs. respective no treatment groups [e.g., vehicle (−IVIg) vs. IVIg; 25 °C vs. 4 °C; no stress (−stress) vs. stress]. *n* = 6 (three experiments with total six mice per group).

The immunosuppression property of IVIg is evident from its ability to ameliorate ITP [19,31,63,64]. We have previously shown that ambient cold exposure inducedimmunosuppression through an IVIg-like pathway in mice, which could be typically induced in wild-type but not in P-selectin deficient (*Selp^−/−^*) mutant mice [18]. Using amelioration on ITP as previously described [18,19], the restraint-stress-induced immunosuppression was demonstrated in mice (Figure 2). Here we found that treatments of IVIg, cold exposure, and restraint stress all ameliorated anti-CD41 Ig injection-induced ITP in wild-type mice (Figure 2A,D,G experiment outline; Figure 2B,E,H). This indicated that restraint stress exerts an immunosuppressive effect as it successfully reduced autoantibody-induced thrombocytopenia (Figure 2H). In agreement with our previous report, such rescue on the ITP cannot be observed in *Selp^−/−^* mutant mice (Figure 2C,F). Intriguingly, here we found that restraint stress can ameliorate ITP in *Selp^−/−^* mice (Figure 2I). Because IVIg and cold exposure exert P-selectin-dependent rescue on ITP, these results suggest that restraint stress exerts immunosuppression through a different pathway, which is independent of P-selectin.

**Figure 3:**
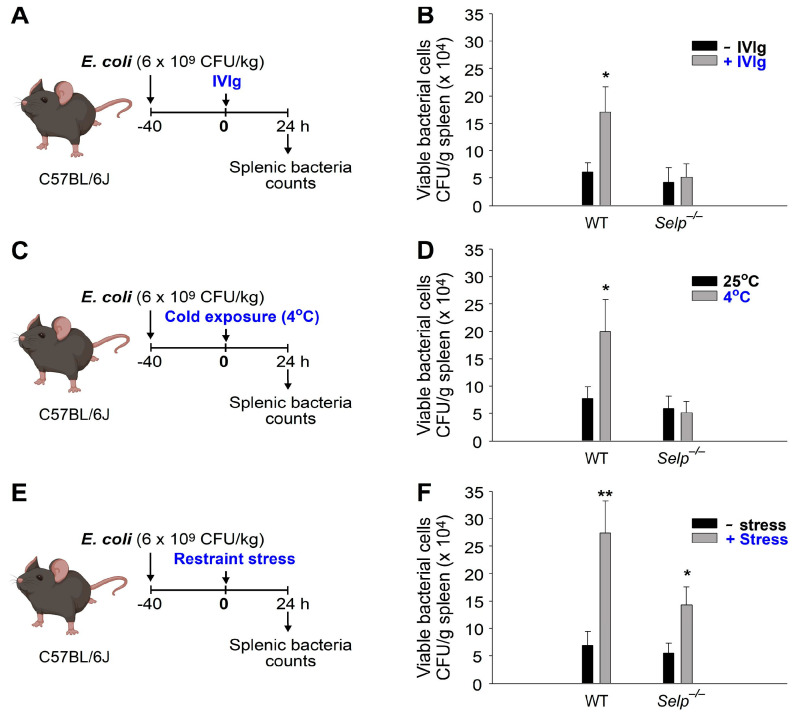
Treatments of IVIg, cold exposure, and restraint-stress-induced immunosuppression as indicated by the suppressed bacterial clearance. (**A**,**B**) IVIg (+IVIg) vs. vehicle (−IVIg) treatments. (**C**,**D**) Cold exposure (4 °C) vs. room temperature (25 °C). (**E**,**F**) Restraint stress (+stress) vs. no stress (−stress). (**A**,**C**,**E**) Experiment outlines. (**B**,**D**,**F**) Viable bacterial levels (CFU/g spleen). Wild-type (WT); P-selectin deficient mice (*Selp^−/−^*). * *p* < 0.05, ** *p* < 0.01, vs. respective no treatment groups [e.g., vehicle (−IVIg) vs. IVIg; 25 °C vs. 4 °C; no stress (−stress) vs. stress]. *n* = 6 (three experiments with total six mice per group).

Evidence has consistently shown that macrophages play a critical role in the phagocytosis and clearance of autoantibody-bound and opsonized platelets in ITP [65,66,67]. Additionally, macrophages are known to play a crucial role in clearing invading bacteria during infections [68,69,70]. Based on these findings, we conducted an analysis of bacterial clearance as described [18], to investigate whether the observed immunosuppressive effects also affect macrophage function (Figure 3). Consistent with the results presented in Figure 2, we discovered that treatments involving IVIg, cold exposure, and restraint stress all suppressed bacterial clearance in wild-type mice (Figure 3A,C,E experimental outline; Figure 3B,D,F WT groups), indicating their immunosuppressive properties. However, such suppression of bacterial clearance was not observed in *Selp^−/−^* mutant mice following IVIg and cold exposure treatments (Figure 3B,D), but it was evident in *Selp^−/−^* mice after 9 h of restraint stress (Figure 3F). These findings strongly suggest that restraint stress induces immunosuppression through a distinct pathway that operates independently of P-selectin.

**Figure 4:**
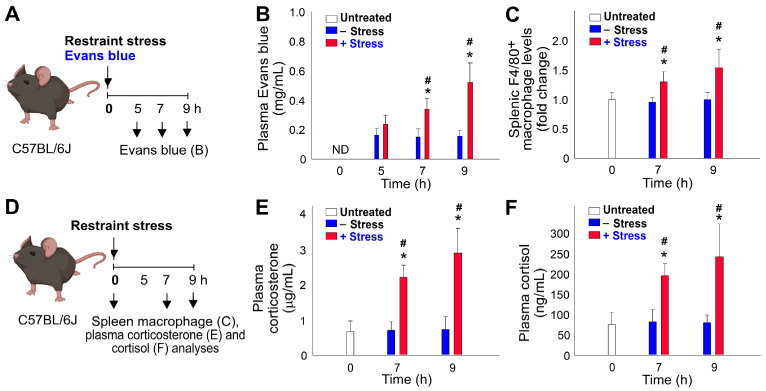
Time-dependent increase in GI leakage, splenic macrophage infiltration, and stress hormone levels in response to the stress in experimental mice. (**A**,**D**) Experimental outlines. (**B**) Plasma levels of Evans blue. (**C**) Levels of F4/80^+^ macrophages in the spleen. (**E**) Plasma corticosterone levels. (**F**) Plasma cortisol levels. * *p* < 0.05, compared to respective 0 h untreated groups; # *p* < 0.05, compared to respective no stress (−stress) groups. The study involved six mice per group, with three experiments conducted in total (*n* = 6).

Here we utilized a previously established mouse model that involved administering Evan blue dye to measure GI leakage levels at various time courses during restraint stress [14,15,16,17]. By employing this Evans-blue-fed mouse model, we made several noteworthy observations. For example, we found a clear kinetic association between restraint-stress-induced GI leakage and increased infiltration of macrophages into the spleen, indicating a potential link between these two phenomena (Figure 4B vs. Figure 4C).

During the restraint stress experiment, we observed the peak levels of all measured parameters at the 9 h mark. Increased GI leakage, splenic macrophage infiltration, and elevated stress hormone levels (corticosterone and cortisol) were most pronounced at this time point, indicating a time-dependent response to restraint stress (Figure 4B,C,E,F).

Furthermore, the infiltration of macrophages into the spleen strongly implies their involvement in the aberrant physiological effects induced by restraint stress. The fact that macrophage infiltration was detected within the spleen, a key organ of the immune system, further supports their potential role in mediating the observed abnormalities triggered by restraint stress (Figure 4C).

### 2.3. Restraint-Stress-Induced Immunosuppression Is Associated with Reduced Phagocyte Function and Increased Cell Death in Mice

**Figure 5:**
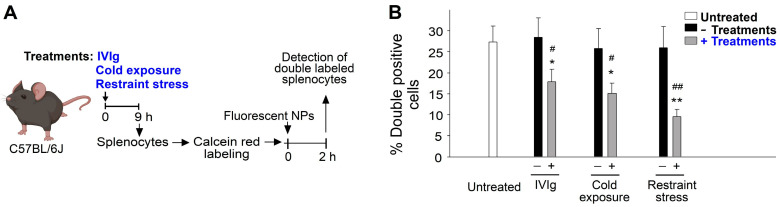
Treatments of IVIg, cold exposure, and restraint stress suppressed the phagocyte function as indicated by the suppression of the engagement between splenic macrophage and fluorescent nanoparticles (NPs). (**A**) Experiment outline. (**B**) Percentage of double-labeled (cell fluorescence + NP fluorescence) cells. * *p* < 0.05, ** *p* < 0.01, vs. respective 0 h groups; # *p* < 0.05, ## *p* < 0.01, vs. respective no treatment groups [e.g., vehicle (−IVIg) vs. IVIg; 25 °C vs. 4 °C; no stress (−stress) vs. stress]. *n* = 6 (three experiments with total six mice per group).

Upon observing relatively high levels of viable bacteria several hours after bacterial injection (Figure 3F) and higher spleen infiltration of macrophages (Figure 4C), we postulated that the impaired phagocyte function of splenic macrophages, specifically their ability to engage and engulf bacteria, might be responsible for restraint-stress-induced immunosuppression. Using described methods [19,71] to assess the phagocyte function of splenic macrophages, we employed fluorescent nanoparticles (NPs) as phagocyte targets. Intriguingly, we discovered that treatments involving IVIg, cold exposure, and restraint stress all resulted in a suppression of spleen phagocyte engagement with fluorescent NPs (Figure 5). Subsequently, to explore the suppressive effect on the live phagocyte population, we examined NP engagements with live splenocytes and observed that restraint stress also suppressed the NP-engaging ability of live splenocytes (Figure S1). These findings strongly suggest that the suppression of phagocyte function contributes to the immunosuppressive effects induced by IVIg, cold exposure, and restraint stress.

**Figure 6:**
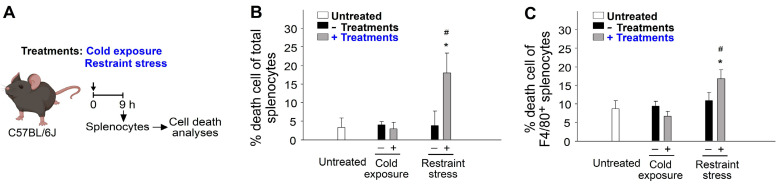
Treatments of IVIg, cold exposure, and restraint stress on the induction of cell death in the population of total splenocytes and splenic macrophages. (**A**) Experiment outline. (**B**) Percentage cell death of total splenocytes. (**C**) Percentage cell death of F4/80^+^ splenic macrophages. * *p* < 0.05, vs. respective 0 h groups; # *p* < 0.05, vs. respective no treatment groups [e.g., 25 °C vs. 4 °C; no stress (−stress) vs. stress]. *n* = 6 (three experiments with total six mice per group).

The induction of cell death in leukocytes has been recognized as a regulatory process associated with immunosuppression [72,73,74]. Additional cell death analyses were therefore conducted and further revealed that restraint stress but not cold exposure induced higher levels of cell death in populations of total splenocytes (Figure 6A, experiment outline, Figure 6B) and F4/80^+^ spleen macrophage (Figure 6C). These results clarify that macrophage cell death is involved in restraint-stress-induced immunosuppressive effects.

### 2.4. Restraint-Stress-Induced Pyroptosis of Spleen Macrophage

**Figure 7:**
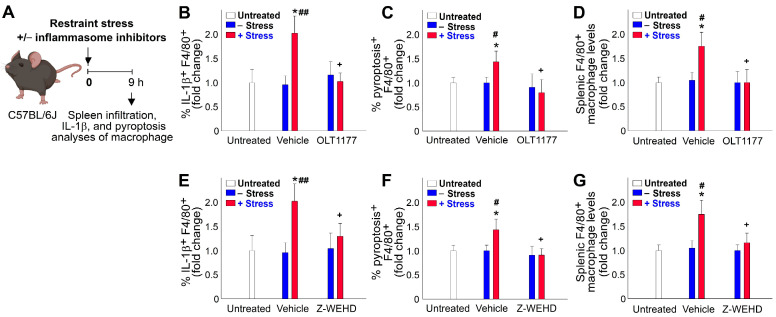
Treatments with NLRP3 inflammasome inhibitors OLT1177 and Z-WEHD effectively rescued restraint-stress-induced spleen infiltration, IL-1β expression, and pyroptotic cell death of spleen macrophages. (**A**) Experiment outline, (**B**–**D**) OLT1177 treatments, and (**E**–**G**) Z-WEHD-FMK (Z-WEHD) treatments are indicated. (**B**,**E**) Relative IL-1β expression levels of F4/80+ spleen macrophages. (**C**,**F**) Relative pyroptosis levels of F4/80^+^ spleen macrophages. (**D**,**G**) Relative macrophage infiltration levels in the mouse spleen. * *p* < 0.05, vs. respective 0 h untreated groups; # *p* < 0.05, vs. ## *p* < 0.01, respective no stress (–stress) groups; + *p* < 0.05, vs. respective vehicle groups. *n* = 6 (three experiments with total six mice per group).

We observed that restraint stress led to GI injury and infiltration of macrophages in the spleen (Figure 4B,C), indicating the potential involvement of an inflammatory response. Here, we examined the expression of the pro-inflammatory cytokine IL-1β in mouse spleen macrophages and found an increased level of IL-1β expression following restraint stress (Figure 7A, experimental outline; Figure 7B,E, vehicle groups). Since inflammasome component caspase-1 activation can trigger IL-1β maturation and pyroptosis, we also assessed the levels of pyroptosis in F4/80^+^ spleen macrophages. Our analysis revealed higher levels of pyroptosis (Figure 7C,F, vehicle groups) and spleen infiltration (Figure 7D,G, vehicle groups) in F4/80^+^ splenic macrophages after restraint stress. To investigate whether the suppression of pyroptosis through treatments with the NLRP3 inflammasome inhibitors OLT1177 and Z-WEHD-FMK could reverse the observed abnormalities induced by restraint stress, we found that OLT1177 and Z-WEHD-FMK treatments both mitigated all the aforementioned pathological responses, including increased pyroptosis, elevated pro-inflammatory IL-1β expression, and spleen infiltration (Figure 7B–G, OLT1177 and Z-WEHD-FMK groups vs. vehicle groups). Although Z-WEHD-FMK primarily inhibits caspase 1, with some inhibition of caspase 4 and 5 to a lesser extent, the combined results from OLT1177 and Z-WEHD-FMK treatments lead us to conclude that such rescue effects are mediated through the NLRP3-caspase 1 axis. Because macrophage cell death was not observed in the ambient cold exposure (Figure 6), these results suggest that restraint stress induces NLRP3-inflammasome-mediated macrophage pyroptosis, which is a notable phenomenon associated with the immunosuppressive effects induced by restraint stress.

## 3. Discussion

The mouse model of restraint stress is a well-established approach utilized to study the physiological, behavioral, and biochemical changes associated with psychological stress in mice [6,7,8]. By subjecting mice to restraint and immobilization stress, researchers can uncover pathophysiological alterations linked to anxiety and stressed behavior in experimental animals [6]. Our analysis indicates that the application of restraint stress induces anxiety-like behavior in mice (Figure 1), suggesting the involvement of psychological stress in the induction of immunosuppression.

IVIg is a therapeutic preparation of pooled human IgG antibodies derived from thousands of healthy donors and has been widely used in various clinical settings for its immunomodulatory properties [18,19]. Apart from its well-established efficacy in treating immune-mediated disorders, accumulating evidence suggests that IVIg can also induce immunosuppressive effects under certain conditions [20,21,22,23,24,25,26,27,28,29]. IVIg-induced immunosuppression has garnered considerable interest, given its potential implications in regulating immune responses and controlling autoimmune and inflammatory diseases [20,21,22,23,24,25,26,27,28,29]. The underlying mechanisms responsible for this immunomodulatory effect are complex and multifaceted, involving interactions with various immune cell populations and immunoregulatory pathways. While IVIg has shown promise in dampening excessive immune responses, the precise factors that determine its immunosuppressive outcomes remain incompletely understood. Previous findings suggested that IVIg’s immunomodulatory properties are dependent on the P-selectin pathway, as evidenced by the lack of rescue effect in ITP observed in P-selectin gene knockout (KO; *Selp*^−/−^) mice [19]. Likewise, ambient cold exposure depends on functional P-selectin and circulating Ig expressions to elicit the anti-inflammatory response, as demonstrated by the loss of immunosuppressive effect in P-selectin knockout (*Selp*^−/−^) and B-cell-deficient (Ig heavy constant mu [*Ighm*]^−/−^) mice [18]. In this present study, we found that restraint stress induces comparable immunosuppressive effects to IVIg treatments and ambient cold exposure. However, restraint-stress-induced immunosuppression does not operate through a P-selectin-dependent mechanism.

These experimental results support the idea that P-selectin may serve as a drug target to treat IVIg and cold-induced immunosuppression. As the immunosuppressive response relies on P-selectin, the administration of P-selectin antagonists may counteract this effect. This hypothesis is reinforced by the observation that immunosuppression is eliminated in mice lacking P-selectin [18,19]. On the other hand, restraint-stress-induced immunosuppression does not rely on the P-selectin pathway. Therefore, using P-selectin antagonists would not be effective in suppressing this type of immunosuppression. However, it is worth noting that P-selectin does play a critical role in protecting against restraint-stress-induced GI leakage, as demonstrated in previous studies [15]. This suggests that although P-selectin is not involved in the induction of immunosuppression, it still plays a significant role in preventing injury associated with restraint stress.

The reasons behind the significant level of immunosuppression induced by restraint stress and the different pathways through which various stressors, such as ambient cold exposure and restraint stress, induce immunosuppression remain unclear. Through the use of an experimental model involving the administration of Evans blue dye to restrained mice [14,15,16,17], we have demonstrated that restraint stress causes gut leakage in mice (Figure 4A, experiment outline, Figure 4B). Since the spleen serves as a key lymphoid organ responsible for filtering blood, including blood from the GI system, to remove pathogens and foreign substances, it is reasonable to observe leukocyte infiltration into the spleen of mice following restraint stress (Figure 4C and Figure 7D). To restore the homeostasis of the immune system, stress hormones may be elicited as a response to restraint stress, leading to the induction of leukocyte cell death. However, the specific underlying mechanisms involved in this process remain largely unknown and warrant further investigation.

Compared to ambient cold exposure, spleen macrophage cell death is a significant observation in this context. Restraint stress induces GI leakage and injuries, leading to pro-inflammatory responses such as IL-1β expression and pyroptosis. The invasion of bacterial components, such as lipopolysaccharide (LPS), due to GI leakage requires macrophages to engulf these compounds to maintain GI homeostasis. Toll-like receptor 4, known as the LPS receptor, has been shown to mediate chronic restraint-stress-induced immune suppression [75], suggesting a potential mechanism that may also apply to acute stress. The role of pyroptosis in restraint-stress-induced immunosuppression is not extensively discussed. A report has indicated that caspase-1 inhibitor VX765 suppresses the production of inflammatory factors, reduces the inflammatory response, and protects the gastric mucosa of mice with acute gastric ulcers induced by cold-restraint stress [76]. This finding is somewhat in agreement with our results, while the detailed mechanism that leads to immunosuppression in acute stress remains elusive.

At the same time, in the context of anti-cancer immune responses, more evidence suggests that pyroptosis plays a role in generating an immunosuppressive effect. For example, it has been shown that pyroptosis and cytokine secretion can promote cancer progression by evading immune surveillance [72]. *Helicobacter pylori* was found to induce pyroptosis, thereby reducing host anti-tumor immunity [73]. Another study highlighted that NLRP3-induced pyroptosis and IL-1β secretion create an immune suppressive environment in breast cancer [74]. These studies shed light on the role of pyroptosis triggered by inflammasomes, which can regulate immunosuppression or immune responses within the tumor microenvironment. Given the elusive nature of the role and detailed mechanism of macrophage pyroptosis in restraint-stress-induced immunosuppression, these findings may provide valuable insights for future studies.

Although valuable results have been obtained in this study, it also unveils several limitations that merit additional attention. For instance, the study effectively demonstrated the impact of restraint stress on anxiety-like behaviors in C57Bl/6J mice. However, the extrapolation of these findings to immune disturbances induced by psychological stress in humans necessitates further investigation. Furthermore, the mechanism underlying the immunosuppression induced by restraint stress diverges from that triggered by IVIg and ambient cold exposure. Nevertheless, this mechanism remains enigmatic and calls for further exploration. Notably, the application of NLRP3 inflammasome inhibitors to suppress pyroptosis and mitigate stress-induced effects unveiled the potential involvement of macrophage pyroptosis in restraint-stress-induced immunosuppression. However, the precise utilization of these inhibitors to counteract stress-induced immunological disruptions remains unclear and warrants deeper investigation. Overall, while the paper adeptly delves into the intricacies of restraint-stress-induced immunosuppression, a more thorough examination of the underlying mechanisms and their broader physiological implications might be necessary to foster a more comprehensive comprehension of the observed phenomenon.

In summary, findings in this report indicate that the mechanism of immunosuppression differs between restraint stress, IVIg, and ambient cold exposure in mice. While IVIg and cold exposure induce immunosuppression through a pathway involving P-selectin, restraint stress exerts immunosuppressive effects through a different pathway independent of P-selectin. The experiments show that three treatments (IVIg, cold exposure, and restraint stress) differentially ameliorate ITP and exacerbate suppressed bacterial clearance in P-selectin-deficient (*Selp^−/−^*) mice, further supporting the involvement of different pathways. Additionally, restraint stress reduces phagocyte function, evidenced by suppressed engagement between splenic macrophages and fluorescent nanoparticles, and leads to increased cell death in total splenocytes and F4/80^+^ splenic macrophages. Furthermore, restraint stress, but not ambient cold exposure, leads to pyroptosis of spleen macrophages, suggesting the potential involvement of macrophage cell death in restraint-stress-induced immunosuppression. Notably, treatments with NLRP3 inhibitors OLT1177 and Z-WEHD-FMK effectively rescue stress-induced macrophage pyroptosis, pro-inflammatory cytokine IL-1β expression, and spleen infiltration. The findings presented here carry important implications for the advancement of treatments aimed at addressing stress-induced immunosuppression. They underscore the necessity for deeper exploration of the underlying mechanisms involved.

## 4. Materials and Methods

### 4.1. Laboratory Mice

Male wild-type C57BL/6J mice aged 8–12 weeks were purchased from the National Laboratory Animal Center (Taipei, Taiwan) [14,15,16,17]. Genetically deficient *Selp*^−/−^ (B6; 129S2-*Selp^tm1Hyn^*/J; P-selectin KO) [47,77,78,79] with a C57BL/6J background were obtained from the Jackson Laboratory (Bar Harbor, ME, USA). *Selp*^−/−^ mice were backcrossed with WT C57Bl/6J mice over six generations. The genotype of the P-selectin KO mice was routinely checked every 15–20 generations following the protocols provided by the Jackson Laboratory. https://www.jax.org/Protocol?stockNumber=002217&protocolID=23572 (accessed on 15 June 2023). All animals were housed in the Animal Center of Tzu-Chi University in a specific pathogen-free, light- and temperature-controlled environment with free access to food and filtered water. Approximately 360 wild-type mice and 100 *Selp*^−/−^ mice were employed. All protocols for examining the experimental animals were approved by the Animal Care and Use Committee of Tzu-Chi University, Hualien, Taiwan (approval ID: 111052).

### 4.2. Open Field Test (OFT) Behavior Test

Using a modified method [50], after 20 h of restraint stress and a subsequent 2 h period of food and water resupply, an OFT behavior test was employed to measure anxiety-, and depression-like behaviors in mice. The test involved using an open-field device, which consisted of a square box measuring 20 cm × 20 cm with surrounding walls that were 30 cm in height. The central area of the arena, measuring 10 cm × 10 cm, was designated as the center zone. Video recordings were captured using an iPhone Xs Max and later analyzed using ToxTrac software (version 2.98). The distance between the mice and the camera during recording was maintained at 60 cm.

### 4.3. Restraint Stress and Restraint-Stress-Induced GI Leakage

An Evans-blue-fed restraint-stress mouse model was employed as described [14,15,16,17]. Mice of ages 10–15 weeks and body weights greater than 26 g were placed in a 50 mL plastic falcon tube for 9 h to induce restraint stress [14,80,81]. To ensure adequate air supply, holes were created at the tapering end of the falcon tube. Blood samples of 50 µL were collected at 0, 5, and 9 h after the stress challenge. Acute restraint stress involved subjecting the mice to a single 5–20 h stress session. In the 5–9 h courses, the experiments were performed during the night (10:00 p.m.–7:00 a.m.), predominantly within their dark cycle or active phase, to minimize the influence of circadian rhythm on the results. Both the no stress and restraint stress groups were deprived of food and water during the restraint stress period. Immediately before the stress challenge began, the mice were administered Evans blue (1.2 g/kg, Santa Cruz Biotechnology, Santa Cruz, CA, USA) via a steel feeding tube [14,15,16,17]. Blood plasma was obtained by collecting blood in an Eppendorf tube and mixing it with an equal amount of anti-coagulant citrate dextrose solution to prevent coagulation [41,42,43]. The collected plasma was then transferred to 96-well plates, and the concentration of Evans blue was measured at 620 nm using a full-spectrum analyzer (Multiskan Spectrum, Thermo Fisher Scientific, Waltham, MA, USA).

### 4.4. Induction and Reversal of ITP

IVIg (Gamimune N) was purchased from Bayer (Whippany, NJ, USA). Experimental ITP was induced as described previously [18,19,82,83]. The mice were intravenously injected with 0.1 mg/kg (body weight) of an antiplatelet monoclonal antibody (rat antimouse integrin α_IIb_/CD41 Ig, clone MWReg30, BD Biosciences, Franklin Lakes, NJ, USA) to induce ITP. To analyze the platelet count, whole blood samples (100–120 μL) of the mice were collected with an anticoagulant, a citrate dextrose solution (38 mM citric acid, 75 mM sodium citrate, and 100 mM dextrose), in Eppendorf tubes. Subsequently, platelet counts were measured using a hematology analyzer (KX-21N, Sysmex, Kobe, Japan) at various time intervals [77,78,84]. To investigate the ameliorative effects of intravenous treatment with IVIg (high dose, 2 g/kg; vehicle: saline; injection at 0 h), cold (4 °C) exposure and restraint stress for 9 h was implemented. Anti-CD41 Ig was treated together with these treatments (IVIg, cold, restraint stress).

### 4.5. Analysis of Bacterial Clearance

The bacterium *E. coli* was cultured using standard methods [85,86,87]. To analyze the immunosuppressive effects of the treatments of IVIg, cold exposure, and restraint stress on bacterial clearance, the experimental mice were challenged by intravenously administering bacteria (*E. coli*, BL21, 6 × 10^9^ CFU/kg; no mortality within 24 h at 4 °C); a 40 h circulating equilibrium at a 25 °C environment was required before treatments of IVIg, cold exposure (control groups: 25 °C exposure), or restraint stress. One day after treatments with IVIg, cold exposure, and restraint stress the spleens of the euthanized mice were collected and weighed. After homogenization (homogenizer, BioSpec Products, Racine, WI, USA) in PBS, surviving bacteria (colony forming units [CFUs]) in the spleen tissues were quantified using the standard plating method.

### 4.6. Measurement of Phagocyte Functions and Cell Death Using Flow Cytometry

Flow cytometers (FACScalibur, BD Biosciences and Gallios, Beckman Coulter Life Sciences) [88,89] were used. Phagocytosis analysis using fluorescent beads by flow cytometer was modified from previously described methods [71,90]. Silica nanoparticles (NPs, 50 nm, 1 mg/mL, Merck/MilliporeSigma, Burlington, MA, USA) [47,91] were opsonized with fluorescein-labeled mouse immunoglobulin (Ig, 10 μg/mL, Jackson ImmunoResearch Laboratories) for 30 min at room temperature (25 °C) in 1× PBS. After blocking with 5% bovine serum albumin (BSA) in 1× PBS for one hour, these NPs (1 × 10^7^) were then mixed with mouse splenocytes (1 × 10^6^; Calcein red labeled) in 200 μL cell-culture medium. After two washes (PBS-albumin, 300 g, 10 min, 25 °C) and fixation (2% paraformaldehyde in PBS, 30 min), the levels of engagement and phagocytosis were revealed by the percentage of NP-bound cells (double-positive), and could be determined using a fluorescence flow cytometer. The IL-1β expression by macrophages was detected using anti-IL-1β antibody (Biolegend, San Diego, CA, USA) and a fluorescent secondary antibody (Jackson Immunoresearch, West Grove, PA, USA). Total death cell and pyroptosis death cell populations were determined using a live/dead cell labeling kit (Zombie NIR™ Fixable Viability Kit, Biolegend; 30 min labeling before all the other staining) [41,43,47] and a pyroptosis labeling kit (Caspase-1 Assay, Green, ImmunoChemistry Technologies, San Jose, CA, USA) [42]. After the cell labeling, the percentage of IL-1β expression, total cell death, and pyroptosis cell death of macrophages could be detected using a flow cytometer. For the inhibition of macrophage NLRP3 inflammasome and caspase-1 activations in mice, we utilized an NLRP3 inflammasome inhibitor, OLT1177 (50 mg/kg) (Cayman Chemical, Ann Arbor, MI, USA), and a caspase-1 inhibitor, Z-WEHD-FMK (10 mg/kg) (R&D Systems, Indianapolis, IN, USA), following previously established protocols [41,42,43,47].

### 4.7. Measurements of Corticosterone and Cortisol

The plasma levels of corticosterone and cortisol were measured using ELISA (corticosterone and cortisol kits: Cayman Chemical).

### 4.8. Statistical Analyses

The experimental results were analyzed using Microsoft Office Excel version 2003 and SPSS 17 and reported as mean ± standard deviation. The statistical significance of the obtained results was examined using one-way analysis of variance and post-hoc Bonferroni-corrected *t* test. A probability of type 1 error α = 0.05 was considered the threshold of statistical significance.

## 5. Conclusions

Our results revealed that restraint stress in C57Bl/6J mice led to anxiety-like behaviors, indicating the involvement of psychological stress. The immunosuppression caused by restraint stress was distinct from that of IVIg and cold exposure, involving reduced phagocyte function and increased spleen macrophage pyroptosis. Treatments with NLRP3 inflammasome inhibitors OLT1177 and Z-WEHD effectively reversed the immunosuppressive effects induced by restraint stress. These findings suggest that macrophage pyroptosis could be a potential therapeutic target for mitigating the impact of stress-induced immunosuppression.

## Data Availability

The datasets used and analyzed during the current study are available from the corresponding author on reasonable request.

## Supplementary Materials

The following supporting information can be downloaded at: https://www.mdpi.com/article/10.3390/ijms241612877/s1.

## References

[1] Segerstrom S.C., Miller G.E. (2004). Psychological stress and the human immune system: A meta-analytic study of 30 years of inquiry. Psychol. Bull..

[2] Marshall G.D. (2011). The adverse effects of psychological stress on immunoregulatory balance: Applications to human inflammatory diseases. Immunol. Allergy Clin. N. Am..

[3] Singh A.K., Chatterjee U., MacDonald C.R., Repasky E.A., Halbreich U. (2021). Psychosocial stress and immunosuppression in cancer: What can we learn from new research?. BJPsych Adv..

[4] Bains J.S., Sharkey K.A. (2022). Stress and immunity—The circuit makes the difference. Nat. Immunol..

[5] Poller W.C., Downey J., Mooslechner A.A., Khan N., Li L., Chan C.T., McAlpine C.S., Xu C., Kahles F., He S. (2022). Brain motor and fear circuits regulate leukocytes during acute stress. Nature.

[6] Campos A.C., Fogaca M.V., Aguiar D.C., Guimaraes F.S. (2013). Animal models of anxiety disorders and stress. Rev. Bras. Psiquiatr..

[7] Pare W.P., Glavin G.B. (1986). Restraint stress in biomedical research: A review. Neurosci. Biobehav. Rev..

[8] Glavin G.B., Pare W.P., Sandbak T., Bakke H.K., Murison R. (1994). Restraint stress in biomedical research: An update. Neurosci. Biobehav. Rev..

[9] Zhang Y., Wu S., Liu Y., Ma J., Li W., Xu X., Wang Y., Luo Y., Cheng K., Zhuang R. (2021). Acute Cold Water-Immersion Restraint Stress Induces Intestinal Injury and Reduces the Diversity of Gut Microbiota in Mice. Front. Cell. Infect. Microbiol..

[10] Rudak P.T., Choi J., Parkins K.M., Summers K.L., Jackson D.N., Foster P.J., Skaro A.I., Leslie K., McAlister V.C., Kuchroo V.K. (2021). Chronic stress physically spares but functionally impairs innate-like invariant T cells. Cell Rep..

[11] Cao M., Huang W., Chen Y., Li G., Liu N., Wu Y., Wang G., Li Q., Kong D., Xue T. (2021). Chronic restraint stress promotes the mobilization and recruitment of myeloid-derived suppressor cells through beta-adrenergic-activated CXCL5-CXCR2-Erk signaling cascades. Int. J. Cancer.

[12] Zhang D., Kishihara K., Wang B., Mizobe K., Kubo C., Nomoto K. (1998). Restraint stress-induced immunosuppression by inhibiting leukocyte migration and Th1 cytokine expression during the intraperitoneal infection of Listeria monocytogenes. J. Neuroimmunol..

[13] Zhang Y., Zhang Y., Miao J., Hanley G., Stuart C., Sun X., Chen T., Yin D. (2008). Chronic restraint stress promotes immune suppression through toll-like receptor 4-mediated phosphoinositide 3-kinase signaling. J. Neuroimmunol..

[14] Chuang D.J., Pethaperumal S., Siwakoti B., Chien H.J., Cheng C.F., Hung S.C., Lien T.S., Sun D.S., Chang H.H. (2021). Activating Transcription Factor 3 Protects against Restraint Stress-Induced Gastrointestinal Injury in Mice. Cells.

[15] Pethaperumal S., Hung S.C., Lien T.S., Sun D.S., Chang H.H. (2022). P-Selectin is a Critical Factor for Platelet-Mediated Protection on Restraint Stress-Induced Gastrointestinal Injury in Mice. Int. J. Mol. Sci..

[16] Sun D.S., Lien T.S., Chang H.H. (2023). Restraint stress-associated gastrointestinal injury and implications from the Evans-blue fed restraint stress mouse model. Tzu Chi Med. J..

[17] Siwakoti B., Lien T.S., Lin Y.Y., Pethaperumal S., Hung S.C., Sun D.S., Cheng C.F., Chang H.H. (2023). The Role of Activating Transcription Factor 3 in Metformin’s Alleviation of Gastrointestinal Injury Induced by Restraint Stress in Mice. Int. J. Mol. Sci..

[18] Chan H., Huang H.S., Sun D.S., Lee C.J., Lien T.S., Chang H.H. (2018). TRPM8 and RAAS-mediated hypertension is critical for cold-induced immunosuppression in mice. Oncotarget.

[19] Huang H.S., Sun D.S., Lien T.S., Chang H.H. (2010). Dendritic cells modulate platelet activity in IVIg-mediated amelioration of ITP in mice. Blood.

[20] Zhang R.L., Lo H.H., Lei C., Ip N., Chen J., Law B.Y. (2020). Current pharmacological intervention and development of targeting IVIG resistance in Kawasaki disease. Curr. Opin. Pharmacol..

[21] Hansen R.J., Balthasar J.P. (2004). Mechanisms of IVIG action in immune thrombocytopenic purpura. Clin. Lab..

[22] Cajamarca-Baron J., Buitrago-Bohorquez J., Mendoza Orozco J.E., Segura O., Guavita-Navarro D., Gallego-Cardona L., Cubides H., Arredondo A.M., Escobar A., Rojas-Villarraga A. (2022). Efficacy and safety of intravenous immunoglobulin in patients with lupus nephritis: A systematic review of the literature. Autoimmun. Rev..

[23] Cornblath D.R., van Doorn P.A., Hartung H.P., Merkies I.S.J., Katzberg H.D., Hinterberger D., Clodi E., ProCID Investigators (2023). Safety and Tolerability of Intravenous Immunoglobulin in Chronic Inflammatory Demyelinating Polyneuropathy: Results of the ProCID Study. Drug Saf..

[24] Pasnoor M., Bril V., Levine T., Trivedi J., Silvestri N.J., Phadnis M., Katzberg H.D., Saperstein D.S., Wolfe G.I., Herbelin L. (2023). Phase 2 trial in acetylcholine receptor antibody-positive myasthenia gravis of transition from intravenous to subcutaneous immunoglobulin: The MGSCIg study. Eur. J. Neurol..

[25] Marcec R., Dodig V.M., Radanovic I., Likic R. (2022). Intravenous immunoglobulin (IVIg) therapy in hospitalised adult COVID-19 patients: A systematic review and meta-analysis. Rev. Med. Virol..

[26] Liu X., Zhang Y., Lu L., Li X., Wu Y., Yang Y., Li T., Cao W. (2023). Benefits of high-dose intravenous immunoglobulin on mortality in patients with severe COVID-19: An updated systematic review and meta-analysis. Front. Immunol..

[27] Lai C.C., Chen W.C., Chen C.Y., Wei Y.F. (2022). The effect of intravenous immunoglobulins on the outcomes of patients with COVID-19: A systematic review and meta-analysis of randomized controlled trials. Expert Rev. Anti-Infective Ther..

[28] Kolahchi Z., Sohrabi H., Ekrami Nasab S., Jelodari Mamaghani H., Keyfari Alamdari M., Rezaei N. (2021). Potential therapeutic approach of intravenous immunoglobulin against COVID-19. Allergy Asthma Clin. Immunol..

[29] Flores-Oria C.A., Saturno E., Ramanathan S., Martinez Castillo D.J., Kumar R., Ferrer N., Mossaad A., Tellez M.E., Jon C., Waters S.C. (2021). Intravenous immunoglobulin as adjuvant therapy for COVID-19: A case report and literature review. SAGE Open Med. Case Rep..

[30] Manganotti P., Garascia G., Furlanis G., Buoite Stella A. (2023). Efficacy of intravenous immunoglobulin (IVIg) on COVID-19-related neurological disorders over the last 2 years: An up-to-date narrative review. Front. Neurosci..

[31] Bayry J., Ahmed E.A., Toscano-Rivero D., Vonniessen N., Genest G., Cohen C.G., Dembele M., Kaveri S.V., Mazer B.D. (2023). Intravenous Immunoglobulin: Mechanism of Action in Autoimmune and Inflammatory Conditions. J. Allergy Clin. Immunol. Pract..

[32] Seeling M., Pohnl M., Kara S., Horstmann N., Riemer C., Wohner M., Liang C., Bruckner C., Eiring P., Werner A. (2023). Immunoglobulin G-dependent inhibition of inflammatory bone remodeling requires pattern recognition receptor Dectin-1. Immunity.

[33] Kindgen-Milles D., Feldt T., Jensen B.E.O., Dimski T., Brandenburger T. (2022). Why the application of IVIG might be beneficial in patients with COVID-19. Lancet Respir. Med..

[34] Tripathi P., Tyagi R.K., Sharma P., Sharma P. (2022). Immunosuppression in Patients with Diabetes Mellitus. Immunosuppression and Immunomodulation.

[35] Berbudi A., Rahmadika N., Tjahjadi A.I., Ruslami R. (2020). Type 2 Diabetes and its Impact on the Immune System. Curr. Diabetes Rev..

[36] Ferracini M., Martins J.O., Campos M.R., Anger D.B., Jancar S. (2010). Impaired phagocytosis by alveolar macrophages from diabetic rats is related to the deficient coupling of LTs to the Fc gamma R signaling cascade. Mol. Immunol..

[37] Pido-Lopez J., Andre R., Benjamin A.C., Ali N., Farag S., Tabrizi S.J., Bates G.P. (2018). In vivo neutralization of the protagonist role of macrophages during the chronic inflammatory stage of Huntington’s disease. Sci. Rep..

[38] Hu Z., Zhan J., Pei G., Zeng R. (2023). Depletion of macrophages with clodronate liposomes partially attenuates renal fibrosis on AKI-CKD transition. Ren. Fail..

[39] Waltl I., Kaufer C., Broer S., Chhatbar C., Ghita L., Gerhauser I., Anjum M., Kalinke U., Loscher W. (2018). Macrophage depletion by liposome-encapsulated clodronate suppresses seizures but not hippocampal damage after acute viral encephalitis. Neurobiol. Dis..

[40] Danenberg H.D., Fishbein I., Gao J., Monkkonen J., Reich R., Gati I., Moerman E., Golomb G. (2002). Macrophage depletion by clodronate-containing liposomes reduces neointimal formation after balloon injury in rats and rabbits. Circulation.

[41] Lien T.S., Sun D.S., Wu C.Y., Chang H.H. (2021). Exposure to Dengue Envelope Protein Domain III Induces Nlrp3 Inflammasome-Dependent Endothelial Dysfunction and Hemorrhage in Mice. Front. Immunol..

[42] Lien T.S., Sun D.S., Hung S.C., Wu W.S., Chang H.H. (2021). Dengue Virus Envelope Protein Domain III Induces Nlrp3 Inflammasome-Dependent NETosis-Mediated Inflammation in Mice. Front. Immunol..

[43] Lien T.S., Chan H., Sun D.S., Wu J.C., Lin Y.Y., Lin G.L., Chang H.H. (2021). Exposure of Platelets to Dengue Virus and Envelope Protein Domain III Induces Nlrp3 Inflammasome-Dependent Platelet Cell Death and Thrombocytopenia in Mice. Front. Immunol..

[44] Tang D., Kang R., Berghe T.V., Vandenabeele P., Kroemer G. (2019). The molecular machinery of regulated cell death. Cell Res..

[45] Bertheloot D., Latz E., Franklin B.S. (2021). Necroptosis, pyroptosis and apoptosis: An intricate game of cell death. Cell Mol. Immunol..

[46] Jin X., Ma Y., Liu D., Huang Y. (2023). Role of pyroptosis in the pathogenesis and treatment of diseases. MedComm.

[47] Hung S.C., Ke L.C., Lien T.S., Huang H.S., Sun D.S., Cheng C.L., Chang H.H. (2022). Nanodiamond-Induced Thrombocytopenia in Mice Involve P-Selectin-Dependent Nlrp3 Inflammasome-Mediated Platelet Aggregation, Pyroptosis and Apoptosis. Front. Immunol..

[48] Blevins H.M., Xu Y., Biby S., Zhang S. (2022). The NLRP3 Inflammasome Pathway: A Review of Mechanisms and Inhibitors for the Treatment of Inflammatory Diseases. Front. Aging Neurosci..

[49] La-Vu M., Tobias B.C., Schuette P.J., Adhikari A. (2020). To Approach or Avoid: An Introductory Overview of the Study of Anxiety Using Rodent Assays. Front. Behav. Neurosci..

[50] Seibenhener M.L., Wooten M.C. (2015). Use of the Open Field Maze to measure locomotor and anxiety-like behavior in mice. J. Vis. Exp..

[51] Shoji H., Miyakawa T. (2020). Differential effects of stress exposure via two types of restraint apparatuses on behavior and plasma corticosterone level in inbred male BALB/cAJcl mice. Neuropsychopharmacol. Rep..

[52] Ma M., Chang X., Wu H. (2021). Animal models of stress and stress-related neurocircuits: A comprehensive review. Stress Brain.

[53] Sanchez-Marin L., Flores-Lopez M., Gavito A.L., Suarez J., Pavon-Moron F.J., de Fonseca F.R., Serrano A. (2022). Repeated Restraint Stress and Binge Alcohol during Adolescence Induce Long-Term Effects on Anxiety-like Behavior and the Expression of the Endocannabinoid System in Male Rats. Biomedicines.

[54] Bak J., Bobula B., Hess G. (2022). Restraint Stress and Repeated Corticosterone Administration Differentially Affect Neuronal Excitability, Synaptic Transmission and 5-HT(7) Receptor Reactivity in the Dorsal Raphe Nucleus of Young Adult Male Rats. Int. J. Mol. Sci..

[55] Xia T.J., Wang Z., Jin S.W., Liu X.M., Liu Y.G., Zhang S.S., Pan R.L., Jiang N., Liao Y.H., Yan M.Z. (2023). Melatonin-related dysfunction in chronic restraint stress triggers sleep disorders in mice. Front. Pharmacol..

[56] Xu Y.X., Liu G.Y., Ji Z.Z., Li Y.Y., Wang Y.L., Wu X.Y., Liu J.L., Ma D.X., Zhong M.K., Gao C.B. (2023). Restraint stress induced anxiety and sleep in mice. Front. Psychiatry.

[57] Mao Y., Xu Y., Yuan X. (2022). Validity of chronic restraint stress for modeling anhedonic-like behavior in rodents: A systematic review and meta-analysis. J. Int. Med. Res..

[58] Chaoui N., Anarghou H., Laaroussi M., Essaidi O., Najimi M., Chigr F. (2022). Long lasting effect of acute restraint stress on behavior and brain anti-oxidative status. AIMS Neurosci..

[59] Li X., Peng Z., Jiang L., Zhang P., Yang P., Yuan Z., Cheng J. (2023). Dlg1 deletion in microglia ameliorates chronic restraint stress induced mice depression-like behavior. Front. Pharmacol..

[60] Afridi R., Suk K. (2023). Microglial Responses to Stress-Induced Depression: Causes and Consequences. Cells.

[61] Lai Z.K., Yin Y.Y., Yan J.Z., Wei Q.Q., Wang B., Li Y.F., Zhang L.M., Wang Y.L. (2023). Inulin-type oligosaccharides of Morinda officinalis exerted antidepressant effects by reducing hippocampal inflammation. Metab. Brain Dis..

[62] Jiang X., Yi S., Liu Q., Su D., Li L., Xiao C., Zhang J. (2022). Asperosaponin VI ameliorates the CMS-induced depressive-like behaviors by inducing a neuroprotective microglial phenotype in hippocampus via PPAR-gamma pathway. J. Neuroinflammation.

[63] Haem S.C. (2022). A single 1 g/kg dose of intravenous immunoglobulin is a safe and effective treatment for immune thrombocytopenia; results of the first HaemSTAR ‘Flash-Mob’ retrospective study incorporating 961 patients. Br. J. Haematol..

[64] Song F., Al-Samkari H. (2021). Management of Adult Patients with Immune Thrombocytopenia (ITP): A Review on Current Guidance and Experience from Clinical Practice. J. Blood Med..

[65] Norris P.A.A., Segel G.B., Burack W.R., Sachs U.J., Lissenberg-Thunnissen S.N., Vidarsson G., Bayat B., Cserti-Gazdewich C.M., Callum J., Lin Y. (2021). FcgammaRI and FcgammaRIII on splenic macrophages mediate phagocytosis of anti-glycoprotein IIb/IIIa autoantibody-opsonized platelets in immune thrombocytopenia. Haematologica.

[66] Swinkels M., Rijkers M., Voorberg J., Vidarsson G., Leebeek F.W.G., Jansen A.J.G. (2018). Emerging Concepts in Immune Thrombocytopenia. Front. Immunol..

[67] Kuwana M., Okazaki Y., Ikeda Y. (2009). Splenic macrophages maintain the anti-platelet autoimmune response via uptake of opsonized platelets in patients with immune thrombocytopenic purpura. J. Thromb. Haemost..

[68] Hirayama D., Iida T., Nakase H. (2017). The Phagocytic Function of Macrophage-Enforcing Innate Immunity and Tissue Homeostasis. Int. J. Mol. Sci..

[69] Pidwill G.R., Gibson J.F., Cole J., Renshaw S.A., Foster S.J. (2020). The Role of Macrophages in Staphylococcus aureus Infection. Front. Immunol..

[70] Jaganathan D., Bruscia E.M., Kopp B.T. (2022). Emerging Concepts in Defective Macrophage Phagocytosis in Cystic Fibrosis. Int. J. Mol. Sci..

[71] Kau J.H., Sun D.S., Huang H.S., Lien T.S., Huang H.H., Lin H.C., Chang H.H. (2010). Sublethal doses of anthrax lethal toxin on the suppression of macrophage phagocytosis. PLoS ONE.

[72] Chauhan D., Vande Walle L., Lamkanfi M. (2020). Therapeutic modulation of inflammasome pathways. Immunol. Rev..

[73] Pachathundikandi S.K., Blaser N., Bruns H., Backert S. (2020). Helicobacter pylori Avoids the Critical Activation of NLRP3 Inflammasome-Mediated Production of Oncogenic Mature IL-1beta in Human Immune Cells. Cancers.

[74] Ershaid N., Sharon Y., Doron H., Raz Y., Shani O., Cohen N., Monteran L., Leider-Trejo L., Ben-Shmuel A., Yassin M. (2019). NLRP3 inflammasome in fibroblasts links tissue damage with inflammation in breast cancer progression and metastasis. Nat. Commun..

[75] Zhang Y., Woodruff M., Zhang Y., Miao J., Hanley G., Stuart C., Zeng X., Prabhakar S., Moorman J., Zhao B. (2008). Toll-like receptor 4 mediates chronic restraint stress-induced immune suppression. J. Neuroimmunol..

[76] Zheng S.Q., Hong X.D., Chen T.S., Luo P.F., Xiao S.C. (2017). Effects of caspase-1 inhibitor VX765 on cold-restraint stress-induced acute gastric ulcer in mice. Chin. J. Burn..

[77] Sun D.S., Ho P.H., Chang H.H. (2016). Soluble P-selectin rescues viper venom-induced mortality through anti-inflammatory properties and PSGL-1 pathway-mediated correction of hemostasis. Sci. Rep..

[78] Sun D.S., Chang Y.W., Kau J.H., Huang H.H., Ho P.H., Tzeng Y.J., Chang H.H. (2017). Soluble P-selectin rescues mice from anthrax lethal toxin-induced mortality through PSGL-1 pathway-mediated correction of hemostasis. Virulence.

[79] Chen J.L., Cheng T.T., Huang C.C., Chang H.H., Lam C.F. (2023). Dual phenotypic characteristics of P-selectin in a mouse model of hemorrhagic shock and hepatectomy. Heliyon.

[80] Zimprich A., Garrett L., Deussing J.M., Wotjak C.T., Fuchs H., Gailus-Durner V., de Angelis M.H., Wurst W., Holter S.M. (2014). A robust and reliable non-invasive test for stress responsivity in mice. Front. Behav. Neurosci..

[81] Chu X., Zhou Y., Hu Z., Lou J., Song W., Li J., Liang X., Chen C., Wang S., Yang B. (2016). 24-hour-restraint stress induces long-term depressive-like phenotypes in mice. Sci. Rep..

[82] Samuelsson A., Towers T.L., Ravetch J.V. (2001). Anti-inflammatory activity of IVIG mediated through the inhibitory Fc receptor. Science.

[83] Sun D.S., King C.C., Huang H.S., Shih Y.L., Lee C.C., Tsai W.J., Yu C.C., Chang H.H. (2007). Antiplatelet autoantibodies elicited by dengue virus non-structural protein 1 cause thrombocytopenia and mortality in mice. J. Thromb. Haemost..

[84] Lien T.S., Sun D.S., Chang C.M., Wu C.Y., Dai M.S., Chan H., Wu W.S., Su S.H., Lin Y.Y., Chang H.H. (2015). Dengue virus and antiplatelet autoantibodies synergistically induce haemorrhage through Nlrp3-inflammasome and FcgammaRIII. Thromb. Haemost..

[85] Sambrook J., Fritsch E.F., Maniatis T. (1989). Molecular Cloning: A Laboratory Manual.

[86] Wong M.S., Chen C.W., Hsieh C.C., Hung S.C., Sun D.S., Chang H.H. (2015). Antibacterial property of Ag nanoparticle-impregnated N-doped titania films under visible light. Sci. Rep..

[87] Sun D.S., Tseng Y.H., Wu W.S., Wong M.S., Chang H.H. (2016). Visible Light-Responsive Platinum-Containing Titania Nanoparticle-Mediated Photocatalysis Induces Nucleotide Insertion, Deletion and Substitution Mutations. Nanomaterials.

[88] Wang T.F., Lin G.L., Chu S.C., Chen C.C., Liou Y.S., Chang H.H., Sun D.S. (2021). AQP0 is a novel surface marker for deciphering abnormal erythropoiesis. Stem Cell Res. Ther..

[89] Chen T.L., Chiang Y.W., Lin G.L., Chang H.H., Lien T.S., Sheh M.H., Sun D.S. (2018). Different effects of granulocyte colony-stimulating factor and erythropoietin on erythropoiesis. Stem Cell Res. Ther..

[90] Liao G., Simone J., Simon S.R. (1994). Paracrine downregulation of Fc gamma RIII in human monocyte-derived macrophages induced by phagocytosis of nonopsonized particles. Blood.

[91] Lien T.S., Sun D.S., Wu W.S., Chang H.H. (2023). Simulation of Hemorrhage Pathogenesis in Mice through Dual Stimulation with Dengue Envelope Protein Domain III-Coated Nanoparticles and Antiplatelet Antibody. Int. J. Mol. Sci..

