# Supplementary material for: Restraint Stress-Induced Immunosuppression Is Associated with Concurrent Macrophage Pyroptosis Cell Death in Mice
Source: Int J Mol Sci. 2023 Aug 17;24(16):12877. doi: 10.3390/ijms241612877 (PMC10454201; doi:10.3390/ijms241612877)
Supplement: Supplementary file 1 [file ijms-24-12877-s001.zip › ijms-2480378-supplementary.pdf]

Supplementary information

Restraint Stress-Induced Immunosuppression Is Associated with Concurrent Macrophage Pyroptosis Cell Death in Mice

Chi-Cheng Li [1,2], Rina Munalisa [3], Hsuan-Yun Lee [3], Te-Sheng Lien [3], Hao Chan [3], Shih-Che Hung [3], Der-Shan Sun [3], Ching-Feng Cheng [4,5] and Hsin-Hou Chang [3,*]

[1] Department of Hematology and Oncology, Buddhist Tzu Chi General Hospital, Hualien 970, Taiwan; kevinlcc1234@gmail.com
[2] Center of Stem Cell & Precision Medicine, Hualien Tzu Chi Hospital, Hualien 970, Taiwan
[3] Department of Molecular Biology and Human Genetics, Tzu-Chi University, Hualien 970, Taiwan; 108727110@gms.tcu.edu.tw (R.M.); 109727102@gms.tcu.edu.tw (H.-Y.L.); alan211@mail.tcu.edu.tw (T.-S.L.); chanhao1011@gmail.com (H.C.); 102353113@gms.tcu.edu.tw (S.-C.H.); dssun@mail.tcu.edu.tw (D.-S.S.)
[4] Department of Pediatrics, Taipei Tzu Chi Hospital, Buddhist Tzu Chi Medical Foundation, Taipei 231, Taiwan; chengcf@mail.tcu.edu.tw
[5] Institute of Biomedical Sciences, Academia Sinica, Taipei 115, Taiwan
* Correspondence: hhchang@mail.tcu.edu.tw; Tel.: +886-3-8565301 (ext. 2667)

Key words: Restraint stress, immunosuppression, macrophage, cell death, pyroptosis, psychological stress, ambient cold exposure, intravenous immunoglobulin

Figures S1: pages 2

Supplemental Figure S1

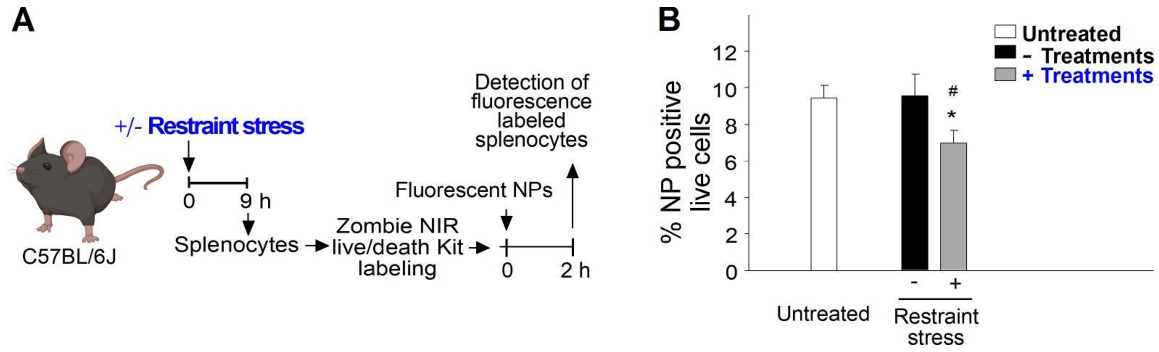

Figure S1. Restraint stress suppressed the live phagocyte function as indicated by the suppression on the engagement between live splenic macrophage and fluorescent nanoparticles (NPs) in mice. (**A**) Experiment outline, and (**B**) percentages of NP-engaged live cells are indicated. Live splenocyte populations of respective groups were normalized to 100 %. * $P < 0.05$, vs. respective 0 h groups (untreated); # $P < 0.05$, vs. respective no stress (−restraint stress) groups. n = 6 (three experiments with total 6 mice per group). Live cell populations were determining using a live/dead cell labeling kit (Zombie NIR™ Fixable Viability Kit, Biolegend).
